# Apolipoprotein D Concentration in Human Plasma during Aging and in Parkinson's Disease: A Cross-Sectional Study

**DOI:** 10.1155/2018/3751516

**Published:** 2018-03-26

**Authors:** Andreas Waldner, Sarah Dassati, Bernhard Redl, Nicola Smania, Marialuisa Gandolfi

**Affiliations:** ^1^Department of Neurological Rehabilitation, Private Clinic Villa Melitta, Via Col di Lana 6, 39100 Bolzano, Italy; ^2^Division of Molecular Biology, Biocenter, Innsbruck Medical University, Innrain 80-82, 6020 Innsbruck, Austria; ^3^Neuromotor and Cognitive Neurorehabilitation Research Centre (CRRNC), Department of Neurosciences, Biomedicine and Movement Sciences, University of Verona UOC Neurorehabilitation—AOUI Verona, University Hospital, P.le L.A. Scuro 10, 37134 Verona, Italy

## Abstract

Apolipoprotein D (ApoD), a lipocalin transporter of small hydrophobic molecules, plays an important role in several neurodegenerative diseases. ApoD is expressed in and secreted from a variety of peripheral and brain tissues. Increments of ApoD have been reported in relation with oxidative stress conditions, aging, and degeneration in the nervous system. Preliminary findings support the role of ApoD in neuroprotection. However, its role in PD remains unclear. To date, no studies have been performed on the relationship between ApoD in the blood and PD, as neurodegenerative pathology related to oxidative damage. We investigated the concentration of ApoD in the blood of healthy control subjects and PD patients with mild-to-moderate neurological impairment. ApoD plasma levels were measured using sandwich enzyme-linked immunosorbent assays (ELISA) in 90 healthy subjects (aging-analysis cohort) and in 66 PD patients at different stages compared with 19 age-matched healthy subjects. Significant age-related increase of ApoD was detected in subjects older than 65 years of age (*p* < 0.002). In PD patients, a significant increase in ApoD plasma concentration was found compared with healthy subjects of the same age (*p* < 0.05). ApoD and PD stage are significantly correlated (*p* < 0.05). ApoD might be a valid marker for the progression of PD.

## 1. Introduction

Aging is the most significant risk factor for neurodegenerative disorders [1]. The progressive age-dependent increase in oxidative stress and inflammation (“inflamm-aging”) represents a conserved and central mechanism of the functional decline occurring in old humans by impairing the dynamic organization of neuronal networks [2, 3]. It is involved in the pathogenesis of several of the major age-related diseases (i.e., cardiovascular disease, type 2 diabetes, and dementia) [4, 5]. The combined age-related induction of both proinflammatory cytokines, tumor necrosis factor *α* (TNF-*α*), and interleukine 6 (IL-6), in parallel with a decrease in antioxidant defence has been suggested to indicate morbidity and mortality in aged individuals [6]. The nervous system (NS) is the central regulator of aging [7]. Parkinson's disease (PD) is the second most common age-related neurodegenerative disorder beside Alzheimer's disease (AD) by affecting 1% of the population older than 65 years [8]. Parkinson's disease is a chronic progressive disease clinically manifested by bradykinesia, rigidity, postural instability, and tremor. Both idiopathic and the rare inherited PD reflect the pathological increase in the oxidative stress and inflammation-related events. The progressive loss of dopaminergic neurons of the substantia nigra (SN) and the presence of Lewy bodies within the same area are the main distinct and neuropathological hallmarks of PD [9]. The resulting dopamine (DA) depletion in their synaptic terminals leads to the cardinal motor symptoms of PD through the formation of dangerous hydroxyl radicals in the presence of ferrous ions [10–12]. The selective dopaminergic neuronal vulnerability is linked to the high iron levels characterizing the SN [11]. Increased levels of oxidized lipids, proteins, and DNA and decreased levels of the antioxidant reduced glutathione were detected in the SN of PD patient [13, 14]. Glutathione reduction is linked to an increased release of the arachidonic acid (AA) via phospholipase A2 followed by an accumulation of reactive oxygen species/reactive nitrogen species (ROS/RNS) and inflammatory mediators [15]. Thus, it has been suggested that limiting AA release and metabolism, a function performed by the atypical lipocalin apolipoprotein D (ApoD), will provide benefit in the early event in the pathogenesis of PD [16, 17].

Apolipoprotein D (ApoD) is a 29 kDa glycoprotein member of the lipocalin family [18]. It comprises an eight-stranded antiparallel *β*-barrel flanked by an *α*-helix. This structure forms a calyx consisting in a hydrophobic ligand pocket that binds AA and progesterone with higher affinity than other small ligands [18, 19]. In humans, ApoD is mainly produced in the plasma and in the brain, in which it is highly expressed in both the white frontal cortex and temporal cortex, in the SN and in the cerebellum. The lowest levels of this lipocalin were found in the hippocampus [20, 21]. In a healthy NS, ApoD is expressed by glia (astrocytes, oligoastrocytes, and Schwann cells) and other nonneuronal cells (e.g., pericytes), and it profoundly affects the function and survival of neurons [18]. In addition to its role in extracellular lipid transport, ApoD modulates AA metabolism in an antioxidant and anti-inflammatory manner [22]. ApoD stabilizes membranes-associated AA by attenuating its release from phospholipids [17]. It traps free AA via sequestration and prevents its consequent conversion into oxidants or into proinflammatory eicosanoids. In addition, ApoD was also demonstrated to reduce hydroperoxides of AA (i.e., eicosatetraenoic acid) to lipid hydroxides, thereby avoiding lipid peroxidation chain reactions and modulating inflammatory pathways [23]. According to its neuroprotective role, ApoD was found to be upregulated in brains affected by neurological (ischemic stroke), neurodegenerative (AD, PD, Niemann-Pick type C disease, transmissible spongiform encephalopathy), and psychiatric disorders (schizophrenia and bipolar disorder). These diseases are characterized by an altered brain AA metabolism and an increased excitotoxicity. Elevated levels of ApoD mRNA and protein were detected in brains after degeneration processes [15]. In contrast to the NS, the expression of ApoD in body fluids was not investigated in much detail during aging. Based on the emerging neuroprotective roles of ApoD the aim of this pilot study was to evaluate (1) ApoD levels in a cohort of healthy controls to verify the effect of aging on ApoD as well as the levels of TNF-*α* in order to get a better insight of the increase in ApoD during the “inflamm-aging” process [2, 24], (2) differences in ApoD levels between PD and age-matched healthy controls, and (3) differences in ApoD levels according to different levels of neurological impairment.

Thus, our pilot study was aimed to validate if ApoD may therefore be a reliable candidate as a marker for PD and/or in the progression of the disease.

## 2. Materials and Methods

### 2.1. Subjects

66 patients with PD (mean age 72.84 ± 7.07 years) and 19 age-matched healthy controls (mean age 72.79 ± 1.55 years) were enrolled. They were white Americans, whose plasma was provided from the Harvard NeuroDiscovery Center Biomarker Study (HBS). They underwent neurological evaluation before enrollment. Inclusion criteria were a diagnosis of PD confirmed according to the United Kingdom Parkinson's Disease Society Brain Bank criteria (bradykinesia, muscular rigidity, rest tremor, and postural instability not caused by primary visual, vestibular, cerebellar, or proprioceptive dysfunction). PD patients were receiving pharmacological therapy (monoamine oxidase-B inhibitors) for PD because of ethical reasons.

Exclusion criteria were any other physiological/pathological factors that can exert an influence on the plasmatic ApoD concentration such as poststroke and/or traumatic brain injury, antipsychotic drugs treatment, obesity (body mass index > 27), idiopathic normal pressure hydrocephalus, Paget's disease, breast cancer, adenocarcinoma of the prostate, glucose- 6-phosphate deficiency, and insulin resistance-related disorders. Patients with PD were subdivided in the following two groups based on their stage of the disease (Hoehn and Yahr classification): 1.0–1.5 (group A, *n*=21) and ≥2.0 (group B, *n*=45).

90 healthy European Caucasian subjects were enrolled (mean age 52.05 ± 19.86 years) from Italy, Austria, and Eastern Europe. They were recruited from among persons who showed no sign of neurological disease and any physiological/pathological factors that can influence ApoD concentration. Healthy controls were subdivided in the following age groups: 20–40 years (*n*=30), 41–65 years (*n*=30), and >65 years (*n*=30).

All participants provided written confirmed consent to participate in the study. The study protocol was approved by the local ethics committee of Gesundheitsbezirk Bozen/Comprensorio Sanitario di Bolzano (Prot 0107531-BZ and Prot 0002995-BZ).

### 2.2. Quantification of Plasma ApoD and TNF-*α* Level

Plasma ApoD and TNF-*α* levels were determined by commercially available enzyme-linked immunosorbent assay kits (USCN Life Science Inc., USA). In brief, human plasma was collected using ethylenediaminetetraacetic acid (EDTA) as an anticoagulant. Samples were centrifuged for 15 minutes at 1000 ×g. Plasma was removed and diluted 1 : 100 with Phosphate Buffered Saline (PBS) (pH = 7.0). Samples were tested in duplicates. 100 *μ*l of each diluted standard, blank, and human samples were precoated with a monoclonal antibody specific to the related molecule (ApoD or TNF-*α*) and incubated for 2 hours. Subsequently, all the samples were incubated for 1 hour with 100 *μ*l/well of a biotin-conjugated polyclonal antibody preparation, washed 3 times with a specific wash solution, and incubated for 30 min with 100 *μ*l avidin conjugated horseradish peroxidase. Following the last 5 washes, 90 *μ*l 3,3′,5,5′-tetramethylbenzidine (TMB) solution was added to each well. The enzyme-substrate reaction was terminated by addition of 50 *μ*l sulphuric acid solution in each well and absorbance was measured spectrophotometrically at a wavelength of 450 nm. The sandwich ELISA used for detection of ApoD, and TNF-*α* had a sensitivity threshold of about 1.03 ng/ml and 0.1 pg/ml, respectively.

### 2.3. Statistical Analysis

Because the data of the 90 Healthy European Caucasian subjects were not normally distributed (Shapiro–Wilk test), nonparametric tests were used for inferential statistics. The Kruskal–Wallis H test (“one-way ANOVA on ranks”) was used to determine if there were statistically significant differences between the three groups of an independent variable (APOD). Post hoc between-group comparisons were performed using the Mann–Whitney *U* test. Since the data of the white Americans (66 PD patients and 19 healthy subjects) were normally distributed (Shapiro–Wilk test), parametric tests were used for inferential statistics. The one-way analysis of variance (ANOVA) was used to determine any statistically significant differences between groups. Post hoc comparisons were performed using Tukey's multiple comparison test to evaluate whether there was any difference between groups after adjusting for multiple testing. Logistic regression was used to predict the probability that PD stage was influenced by ApoD levels. Age, gender, and PD medications were considered as covariates. Statistical analyses were carried out using the SPSS^®^ Statistics version 20.0. *p* < 0.05 was set as a significant value for the first level of analysis.

Statistical analyses were carried out using the SPSS Statistics version 20.0. *p* < 0.05 was set as a significant value for the first level of analysis.

## 3. Results

### 3.1. Relationship between ApoD and Aging

**Figure 1:**
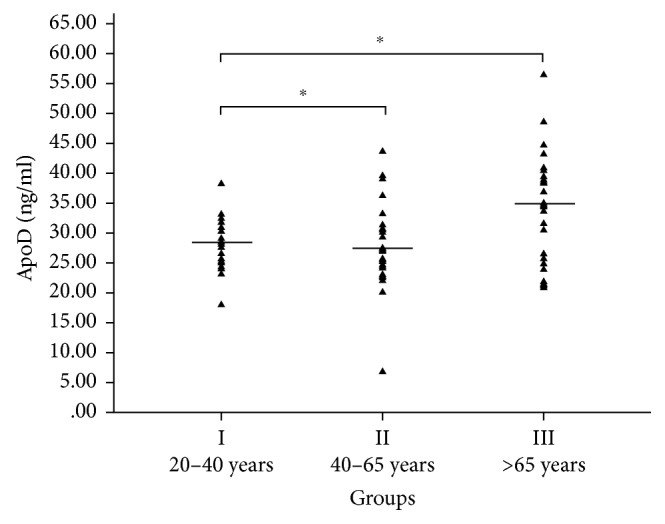
Age-dependent ApoD concentration in human plasma. Horizontal lines show the average distribution of age-related ApoD concentration in plasma in group I (mean 28.28 ± 4.01 ng/ml, *n*=30, and 20–40 years), group II (mean 27.73 ± 7.26 ng/ml, *n*=30, and 40–65 years), and group III (mean 34.61 ± 9.21 ng/ml, *n*=30, and >65 years). The increase in ApoD concentration was significant in subjects of group III compared to subjects of group I (*p* < 0.001) and group II (*p* < 0.002).

Age-related ApoD levels in the plasma of healthy subjects are shown in Figure 1. The reported data were referred to subjects subdivided in different age groups: 20–40 years (group I, *n*=30), 41–65 years (group II, *n*=30), and >65 years (group III, *n*=30). Increased ApoD levels were found only in older subjects aged >65 years, independently of gender. As shown in Figure 1, the increase in ApoD concentration was significant in subjects of group III (mean 34.61 ± 9.21 ng/ml) compared with subjects of group I (mean 28.28 ± 4.01 ng/ml) (*p* < 0.001) and group II (mean 27.73 ± 7.26 ng/ml) (*p* < 0.002). ApoD concentration did not significantly increase in subjects of group II (mean 27.73 ± 7.26 ng/ml) compared with younger subjects of group I (mean 28.28 ± 4.01 ng/ml) (*p* > 0.5). Thus, age-related ApoD concentration in human plasma increases significantly in older subjects >65 years.  Table 1 shows the gender-unrelated and age-dependent ApoD increase in human plasma of healthy European Caucasian subjects.

### 3.2. Relationship between TNF-*α* and Aging

**Figure 2:**
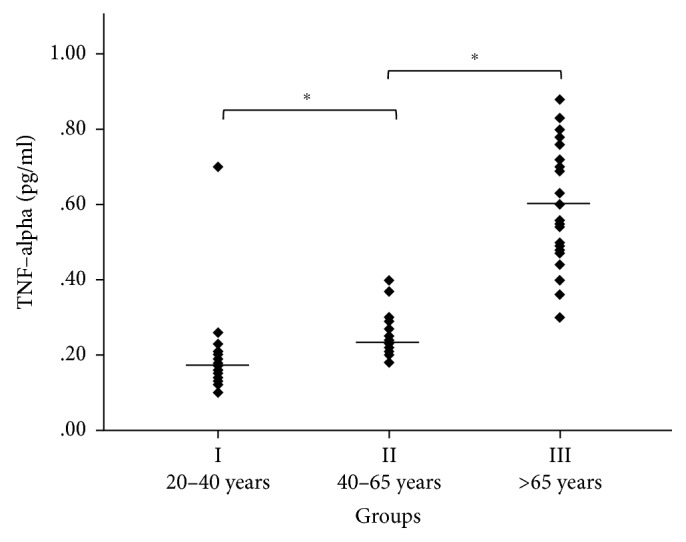
Age-dependent TNF-*α* concentration in human plasma. One-way analysis of variance (ANOVA) and Student's *t*-test with Tukey's post hoc were used. Horizontal lines show the average distribution of age-related ApoD concentration in human plasma in group I (mean 0.188 ± 0.1 pg/ml, *n*=30, and 20–40 years), group II (mean 0.24 ± 0.05 pg/ml, *n*=30, and 40–65 years), and group III (0.61 ± 0.15 pg/ml, *n*=30, and >65 years). The increase in TNF-*α* concentration in human plasma was significant between groups I and II (*p* < 0.05) and between groups II and III (*p* < 0.0001).

As shown in Figure 2, we found the following TNF-*α* concentrations: 0.188 ± 0.1 pg/ml in group I, 0.24 ± 0.05 pg/ml in group II, and 0.61 ± 0.15 pg/ml in group III. Thus, the increase in TNF-*α* concentration in human plasma was significant between groups I and II (*p* < 0.5) and between groups II and III (*p* < 0.0001). In agreement with a previous study, TNF-*α* levels in human plasma of European Caucasian subjects during aging were found to be gender-unrelated in our study (Table 1) [6].

### 3.3. Relationship between ApoD and Disease Severity in Parkinson's Disease

**Figure 3:**
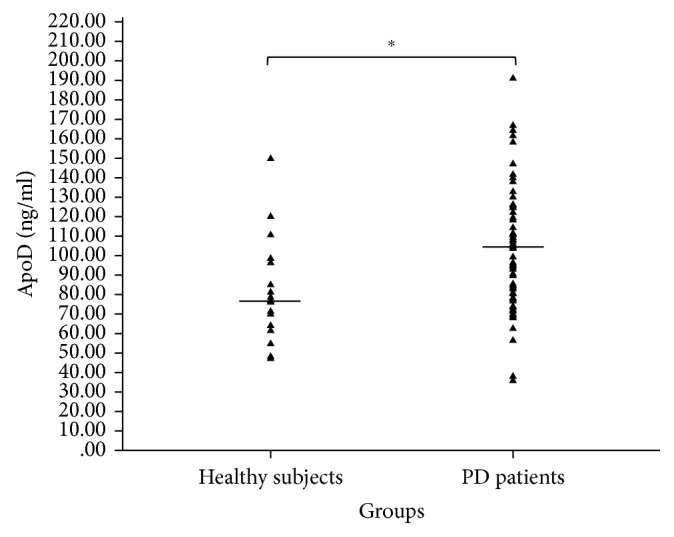
Plasma ApoD levels in healthy subjects and in PD.

**Figure 4:**
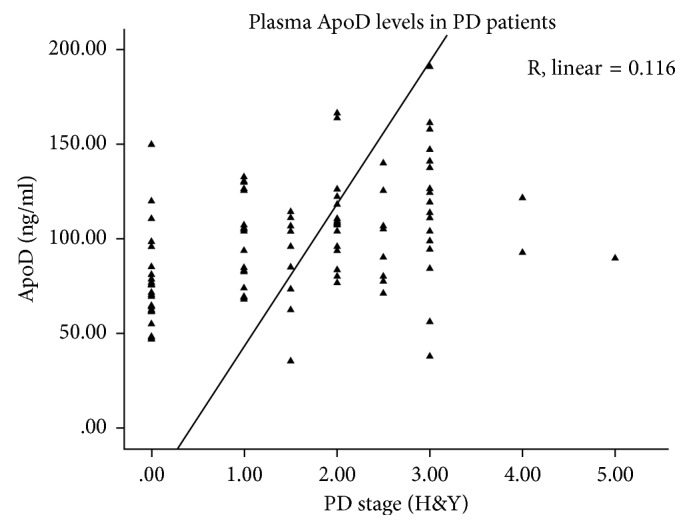
Plasma ApoD levels correlate with disease severity in PD. The linear regression analysis adjusted for age, gender, and PD medications reveal an increase in the plasmatic levels of ApoD, which is correlated with disease severity in PD (*p* < 0.01). Control data were included in the regression analysis as PD stage 0.

ApoD levels were compared among control and PD subjects, who were in stages 1.0 to 5.0 in the scale of Hoehn and Yahr (H&Y). Plasmatic ApoD levels were higher in all PD patients (mean 104.15 ± 30.96 ng/ml) compared with age-matched healthy subjects (mean 79.35 ± 26.25 ng/ml) (*p* < 0.005) (Figure 3). PD subjects were subdivided in the following groups (Hoehn and Yahr classification): 1.0–1.5 (group A, *n*=21) and ≥2.0 (group B, *n*=45). Increased values of ApoD concentration were detected in PD patients of group B (mean 109.10 ± 32.31 ng/ml) (*p* < 0.005) compared with the healthy subjects. No difference was found between the 2 groups of patients. Stage and ApoD were significantly correlated (*p* < 0.05; Spearman's *r*=0.116). Thus, ApoD is predictive of the stage (linear regression model) (Figure 4). ApoD levels in human plasma in PD were found to be gender-unrelated in human plasma of the white Americans group (Table 2).

## 4. Discussion

In the current study, we first investigated if and how “inflamm-aging” influences ApoD concentration in human plasma. The plasmatic increase in ApoD concentration found in subjects older than >65 years supports our hypothesis. The detected velocity in ApoD increase between group II and III reflects the age-related overproduction of the ROS/RNS and inflammatory factors (*p* < 0.002). The plasmatic levels of ApoD start to increase at the age-related baseline upon which the entity of “inflamm-aging” slowly overwhelms the endogenous antioxidant and anti-inflammatory response. It would be hypothesized that “inflammatory tone” is observed to start rising before (in the group II age range) and that the increase in ApoD takes place later. This is highlighted by the significant increase in TNF-*α* concentration between the same groups (*p* < 0.0001). In addition, the incidence and prevalence of AD, which double every 5 years after an age of 65, contribute to the concept of the age-related baseline discussed above [24, 25]. Our findings are in agreement with the emerging role of ApoD as anti-stress protein in aging [15]. In a cross-species comparative analysis of transcription changes during brain aging, ApoD was identified as the most upregulated gene in the aged brain [3]. Overexpression of the human APOD gene increased survival under stress in the mouse and extended the mean lifespan in *Drosophila melanogaster* [26, 27]. The ApoD knockout (ApoD-KO) mouse model is characterized by prematurely aged, hyperkinesia and memory deficits. The loss of ApoD was found to affect the age-dependent transcriptome patterns of the cortex and hippocampus [28]. By contributing to the adaptability and plasticity occurring against the age-related morphological atrophic changes, ApoD protects the integrity of the NS by preserving organisms against the deleterious effects of aging [15, 27, 28]. In conclusion, the results of our pilot study provide the first evidence of an age-dependent increase of ApoD concentration in human blood plasma. In addition, they highlight the potential utility of ApoD as a promising candidate as a marker of the progressive inability to counterbalance the oxidative stress-inflammation-related insults by endogenous antioxidant defence.

Increasing evidences highlight the involvement of both oxidative stress and inflammation in the deregulation of plasma membrane lipid metabolism in PD and other neurodegenerative disorders [5, 14, 29, 30]. Specifically, an upregulation of the AA metabolic cascade was found in brain regions with a chronic SN lesion [31]. Consequently, a significant increase in ApoD expression was shown in glial cells in postmortem human brains of PD patients [32]. Thus, we investigated if and how ApoD concentration changes in human plasma of PD patients at different stages of their disease. We detected a higher increase of ApoD in PD patients compared to age-matched healthy subjects (*p* < 0.005). Increased values of ApoD concentration were found only in PD patients of the group B (*p* < 0.005). Stage and ApoD were significantly correlated (*p* < 0.05; Spearman's *r*=0.116). Our findings are in agreement with the emerging role of ApoD in protecting cells against astrogliosis, whose increase contributes to the deterioration of motor symptoms of PD. By being required to compensate the loss of dopaminergic neurons as well as to control the spreading of consequent damages, reactive astrocytes can become dangerous once out of control. Thus, astrogliosis is a “double-edged sword.” A role for ApoD in maintaining the glial response against the plethora of ROS/RNS and inflammatory mediators under fixed limits has been proposed [15, 33, 34]. The role of ApoD in modulating astrocyte reactivity has been well demonstrated both in mouse primary glial cultures and *in vivo* in controlling the on-off signals that tune the glial response to injury [33]. In addition, ApoD might be involved in removal of neurotoxic molecules released during cell dying [35]. ApoD has been also shown to contribute to an autocrine-protecting mechanism in astrocytes, avoiding accumulation of peroxidated lipids and altering the paraquat transcriptional response of genes involved in ROS managing and the inflammatory response to OS [30]. ApoD acts as a lipid transporter protein in neurite-promoting responses, in active myelination and in axonal outgrowth as well as a regeneration-promoting agent [33–37]. In addition, ApoD exerts its neuroprotective role by repairing the damaged lysosomal membranes. The pH recovery in PQ-treated lysosomes is mediated by ApoD [38]. Thus, all the emerging evidences supporting a central role of ApoD in PD-associated astrogliosis can explain our detected correlation of plasma ApoD levels with disease severity in PD. Given this perspective, new strategies aimed to augment ApoD neuroprotective properties together with therapeutic approaches targeting CNS-resident immune cells such as microglia and mast cells [15, 39]. All the patients enrolled in the study were receiving pharmacological therapy for PD because of ethical reasons. It has not been reported an influence of the different types of dopaminergic agents on the expression of ApoD, whereas an increase in AA and a decrease in docosahexaenoic acid (DHA) concentrations were found in postmortem brain in PD patients and parkinsonian monkeys treated with levodopa [40]. Consequently, a possible influence of levodopa on the detected ApoD levels cannot be excluded. In addition, a potential reduction in ApoD levels exerted from the antioxidant effects of monoamine oxidase (MAO) inhibitors has to be considered. These drugs reduced hyperactivity of MAO, which leads to an excessive production of hydrogen peroxide involved in neurotoxicity [41]. Interestingly, the combination of MAO inhibitors and levodopa was found to improve cognition, including probabilistic learning, working memory, and executive functions [42]. The potential contribution of ApoD as a lipid carrier in these drugs-related remodelling processes within the glial-neuronal networks has to be investigated. This is the first study of plasma ApoD in relation to PD and its progression. The strengths of the present study are the large patient sample for a preliminary study and the evaluation of effects within the brain by detecting changes in blood, a less intrusive procedure to collect from human subjects. Its limitation is the possibility that differences between TNF-*α* and ApoD concentration might also be the reflection of different tissue sources producing TNF-*α* (i.e., age). In conclusion, we found a significant increase in the plasmatic levels of ApoD in PD, which are correlated with disease severity (*p* < 0.05; Spearman's *r*=0.001). Our study supports the idea that measuring the concentration of ApoD in human plasma may be used as a reliable marker for the progression of PD.

## 5. Conclusion

This study suggests a consistent age-related physiological increase of ApoD concentration in human plasma, especially in subjects over an age of 65 years. Although there is a general age-related increase in ApoD concentration during aging, we found that plasma levels of PD patients are significantly higher than age-matched healthy subjects. Parkinson's disease stage and ApoD are significantly correlated. The increase in the plasmatic levels of ApoD in PD is significant even in the presence of comorbidities, potentially compromising the lipid homeostasis as, for example, in hyperlipidemia. Considering the sample limitations and exploratory nature of this pilot study, further studies with larger sample size of healthy subjects and PD patients are recommended in order to verify the validity of ApoD that may therefore be a reliable candidate as a marker for disease progression.

## Figures and Tables

**Table 1:** Gender distribution of age-dependent ApoD and TNF-*α* levels in human plasma.

Group	Age (years)	ApoD concentration (ng/ml)			TNF-*α* concentration (pg/ml)		
		Females	Males	Total	Females	Males	Total
I	20–40	27.40 ± 4.17 (*n*=14)	28.75 ± 2.85 (*n*=16)	28.28 ± 4.01 (*n*=30**)**	0.17 ± 0.02 (*n*=14)	0.20 ± 0.13 (*n*=16)	0.188 ± 0.1 (*n*=30**)**
II	40–65	27.85 ± 5.77 (*n*=14)	27.65 ± 8.55 (*n*=16)	27.73 ± 7.26^∗^(*n*=30)	0.25 ± 0.05 (*n*=13)	0.24 ± 0.05 (*n*=15)	0.24 ± 0.05^∗^ (*n*=28)
III	>65	31.37 ± 9.71 (*n*=24)	34.65 ± 7.45 (*n*=6)	34.61 ± 9.21^∗^ (*n*=30)	0.65 ± 0.13 (*n*=24)	0.62 ± 0.14 (*n*=4)	0.61 ± 0.15^∗^ (*n*=28**)**

^∗^ *p* < 0.05.

**Table 2:** Gender distribution of ApoD levels in human plasma in PD.

Group	H&Y	Age (years)	ApoD (ng/ml)		
			Females	Males	Total
A	<2	71.57 ± 5.24	90.18 ± 25.70 (*n*=15)	104.31 ± 17.90 (*n*=6)	93.54 ± 24.47 (*n*=21)
B	≥2	73.44 ± 8.99	107.67 ± 39.83 (*n*=21)	111.81 ± 26.59 (*n*=24)	109.10 ± 32.31 (*n*=45)
All PD	1–5	72.84 ± 7.07	99.90 ± 34.63 (*n*=38)	110.47 ± 25.25 (*n*=28)	104.15 ± 30.96 (*n*=66)
Healthy control group	72.79 ± 1.55	85.33 ± 24.32 (*n*=11)	90.26 ± 14.29 (*n*=8)	79.35 ± 26.25 (*n*=19)	

^∗^ *p* < 0.05.
